# Deliver Anti-inflammatory Drug Baicalein to Macrophages by Using a Crystallization Strategy

**DOI:** 10.3389/fchem.2020.00787

**Published:** 2020-09-11

**Authors:** Jianming Zhang, Chao Teng, Caolong Li, Wei He

**Affiliations:** ^1^Shanghai Mental Health Center, Shanghai Jiao Tong University School of Medicine, Shanghai, China; ^2^School of Pharmacy, China Pharmaceutical University, Nanjing, China; ^3^School of Science, China Pharmaceutical University, Nanjing, China; ^4^Shanghai Skin Disease Hospital, Tongji University School of Medicine, Shanghai, China

**Keywords:** anti-inflammation, baicalein, macrophages, nanocrystals, low solubility

## Abstract

Macrophages are potent to modulate inflammation *via* phenotypic switch and production of inflammatory factors. Baicalein (BCL) is frequently used to alleviate inflammation; however, its application is always hindered due to low solubility. Herein, BCL nanocrystals (BNRs) were prepared to improve its delivery to macrophages. The prepared BNRs have a diameter of 150 nm with a rod-like structure. The nanocrystals could be well-taken up by macrophages *via* the caveolar pathway and, therefore, promote the polarization switch from proinflammatory phenotype to anti-inflammatory macrophages and alleviate the inflammation *via* reducing production cytokine IL-12. In conclusion, the crystallization strategy is promising for the improvement of the solubility of BCL and promotion of its anti-inflammatory activities.

## Introduction

Inflammation is an important life activity, protecting the body from infection with foreign organisms; however, chronic inflammation always results in exacerbation of numerous diseases such as cardiovascular diseases, arthritis, and inflammatory bowel disease (He W. et al., 2019). Macrophages play a central role in the regulation of inflammation *via* phenotypic switch and production of inflammatory factors (Leitinger and Schulman, 2013).

Baicalein (BCL), a major flavonoid of *Scutellaria baicalensis*, is a potent anti-inflammatory agent predominantly acting through associating with a variety of chemokines including IL-8, macrophage inflammatory protein (MIP)-1β, and monocyte chemotactic protein (MCP)-2 and limiting their biological function (Li et al., 2000). Moreover, baicalein can polarize proinflammatory phenotype M1 to anti-inflammatory M2 and alleviate inflammatory response (Zhu et al., 2016). As a result, BCL has a promising potential to combat inflammation. However, its use is always limited due to low solubility in water (Zhang et al., 2009). Nanotechnology, such as liposomes, nanoemulsions, polymeric micelles, and nanocrystals, is potent to enhance the solubilization of poorly water-soluble active compounds, along with over 65 products approved for clinical use (Anselmo and Mitragotri, 2016; He H. et al., 2019; He et al., 2020; Yu et al., 2020). Of them, nanocrystal technology prepared by formulating drug particles into nanosized particles by top-down or bottom-up approaches is one of the most promising means to solve the solubility (Lu et al., 2019), owing to its advantages including its high drug loading, enhanced dissolution rate and saturation solubility, reproducibility of oral absorption, improved dose-bioavailability proportionality, and increased patient compliance (He et al., 2013, 2016; Lv et al., 2018; Zhou et al., 2018; Zhao et al., 2019). So far, over 15 nanocrystal formulations such as Rapamune®, Emend®, Tricor®, Megace ES®, Avinza®, Focalin XR®, Ritalin®, and Zanaflex Capsules™ have been approved. In particular, the formulation of nanocrystals has an extremely high drug-loading capacity that is markedly greater than that from conventional nanocarriers with the capacity of <10% (He et al., 2013; Anselmo and Mitragotri, 2019). Accordingly, nanocrystal technology has a potential to address the solubility of BCL and improve its delivery.

In this study, BCL nanocrystals (BNRs) were prepared and characterized. Moreover, the uptake and internalization pathway in macrophages, phenotypic switch, and anti-inflammation *in vitro* were investigated, and finally, biocompatibility of BNRs was evaluated.

## Materials and Methods

### Materials

Beta-lactoglobulin (β-LG), fluorescein isothiocyanate isomer I (FITC), rhodamine B isothiocyanate (RITC), 3-(4,5-dimethylthiazol-2yl)-2,5-diphenyltetrazoliumbromide (MTT), and polyethylenimine (PEI, 408727, 25,000 Da) were provided by Sigma-Aldrich Co., Ltd. (St. Louis, MO, USA). Fetal bovine serum (FBS), Dulbecco's Modified Eagle Medium (DMEM), and trypsin were from Thermo Fisher Scientific, Inc. (Waltham, MA, USA). 4,6-Diamino-2-phenyl indole (DAPI) were brought from the Beyotime Institute of Biotechnology (Haimen, China). Nystain and methyl-β-cyclodextrin (M-CD) were supplied by Aladdin Co., Ltd. (Aladdin Co., Ltd., Shanghai, China). BCL was obtained from Chengdu Pufei De Biotech Co., Ltd. (Chengdu, China). Antibodies of CD206 and INOS were obtained from Proteintech Co., Ltd. (Rosemont, USA). Cy5 was obtained from Solarbio Science & Technology Co., Ltd. (Shanghai, China). Alexa Fluor® 488-Cave-1/F-actin/CTB was from Abcam Trading Co., Ltd. (Shanghai, China). ELISA kits of mouse interferon-α (INF-α) and interleukin-12 (IL-12) were purchased from Enzyme-linked Biotechnology Co. Ltd. (Shanghai, China).

### Cell Cultures and Animals

RAW 264.7 cells were cultured in DMEM containing 10% FBS and 1% penicillin/streptomycin at 37°C, 5% CO_2_, and 100% humidity and were split when confluent. Before use, the cells were polarized into M1 phenotype by incubation with lipopolysaccharides (100 ng/mL) and interferon-γ (20 ng/mL) for 24 h.

The animals used in all experiments received care in compliance with the Principles of Laboratory Animal Care and the Guide for the Care and Use of Laboratory Animals. Animal experiments followed a protocol approved by the China Pharmaceutical University Institutional Animal Care and Use Committee.

### Preparation and Characterization of Baicalein Nanocrystals

The BCL nanocrystals (BNRs) were prepared by the precipitation–ultrasonication method with a cationic beta-lactoglobulin (CLG) as a stabilizer (He et al., 2013; Xin et al., 2019). Briefly, BCL dissolved in 1 mL dimethyl sulfoxide (DMSO) was mixed with 10 mL CLG solution (1 mg/mL) under stirring and treated with an ultrasonic probe (20–25 kHz, Scientz Biotechnology Co., Ltd., Ningbo, China) at 300 W for 10 min in an ice bath. Dye-labeled nanoparticles were prepared by a similar method except for dissolving the dye and the drug in DMSO together in advance for the mixing.

Serum stability of nanoparticles was performed by incubation in PBS containing 10% FBS at 37°C. At specific time points, the particle size was tested by using a 90Plus Particle Size Analyzer (Brookhaven Instruments, Holtsville, NY) at 25°C.

The nanoparticles' shape was tested by a transmission electron microscope (TEM, JEM-1230, Tokyo, Japan) under an acceleration voltage of 200 kV. In brief, one drop of diluted sample was placed onto the copper mesh, and then the mesh was dried, stained with 2% (w/w) phosphotungstic acid for 30 s, and finally dried at 25°C for 5 min.

The coating of the stabilizer on the nanoparticles was studied by fluorescence resonance energy transfer (FRET) in a fluorescence spectrometer (SHIMADZU RF-5301PC, Japan), in which FITC and RITC were utilized as the energy donors and acceptors. The scanning was performed at an excitation wavelength of 492 nm. The split width was 5 nm for the excitation and 15 nm for the emission.

### *In vitro* Drug Release

The drug release was investigated by a dialysis method. In brief, the samples were added into a dialysis bag (3,500 Da) and immersed in a shaking incubator (SHA-C, Jintan, China) with a speed of 100 rpm/min at 37°C. At pre-determined time intervals, samples were collected and purified with a 0.2 μm filter. The drug content was assayed in a high-performance liquid chromatography system equipped with an ultraviolet detector (SHIMAZU LC-10AT, Kyoto, Japan). The separation was performed on the ODS C18 column (250 mm × 4.6 mm, Diamonsil, Beijing, China) at 30°C at 276 nm. The mobile phase consisted of methanol and 0.05% phosphoric acid (70/30, v/v) and was pumped at a flow rate of 1 mL/min (Teng et al., 2020).

### Cell Experiments

Cells cultured on the 6-well plates (1 × 10^5^ cells/well) were incubated with the samples for 4 h and then were subjected to determination by flow cytometry (BD FACS Calibur, San Jose, CA, USA) and observation by using confocal laser scanning microscopy (CLSM, LSM700, Carl Zeiss, Germany). To study the internalization pathway, the cells were pre-incubated with inhibitors, nystatin (10 mM), or M-CD (2.5 mM), for 0.5 h. The colocalization was examined by confocal microscopy after incubation with the nanoparticles and staining with Alexa Fluor® 488-Cave-1, -F-actin, or -CTB for 3 h.

The polarization of RAW 264.7 cells were assayed by CLSM and flow cytometry. Briefly, the cells (1 × 10^5^ cells/well) were cultured with the preparations for 12 h, incubated with a primary and secondary antibody for 2 and 24 h at 4°C, respectively, and stained with DAPI for 15 min. The anti-inflammation *in vitro* was tested by detecting IL-12 with ELISA kits after a 12 h incubation with various preparations. Cell viability was studied by MTT assay after incubation with preparations for 24 h.

### Biocompatibility Study

Hemolytic activity was studied by incubating the formulations with erythrocytes. In brief, erythrocytes were collected from mouse blood by centrifugation at 1,500 × g for 15 min, wash, and resuspension with PBS. Then, CLG or PEI was incubated with the erythrocytes at 37°C for 1 h, followed by centrifugation at 1,500 × g for 15 min, collection of the supernatant, and comparison of hemolytic activity.

The immunogenicity was assessed by detecting IL-12 and INF-α in the plasma with ELISA kits at pre-determined time intervals after intravenous injection of various preparations at specific doses according to the body weight of mouse.

### Statistical Analysis

One-way analysis of variance was performed to assess the statistical significance of the differences between samples. The results are expressed as the mean ± standard deviation (SD). *P* < 0.05 indicated significant differences.

## Results and Discussion

### Preparation and Characterization of BNRs

**Figure 1:**
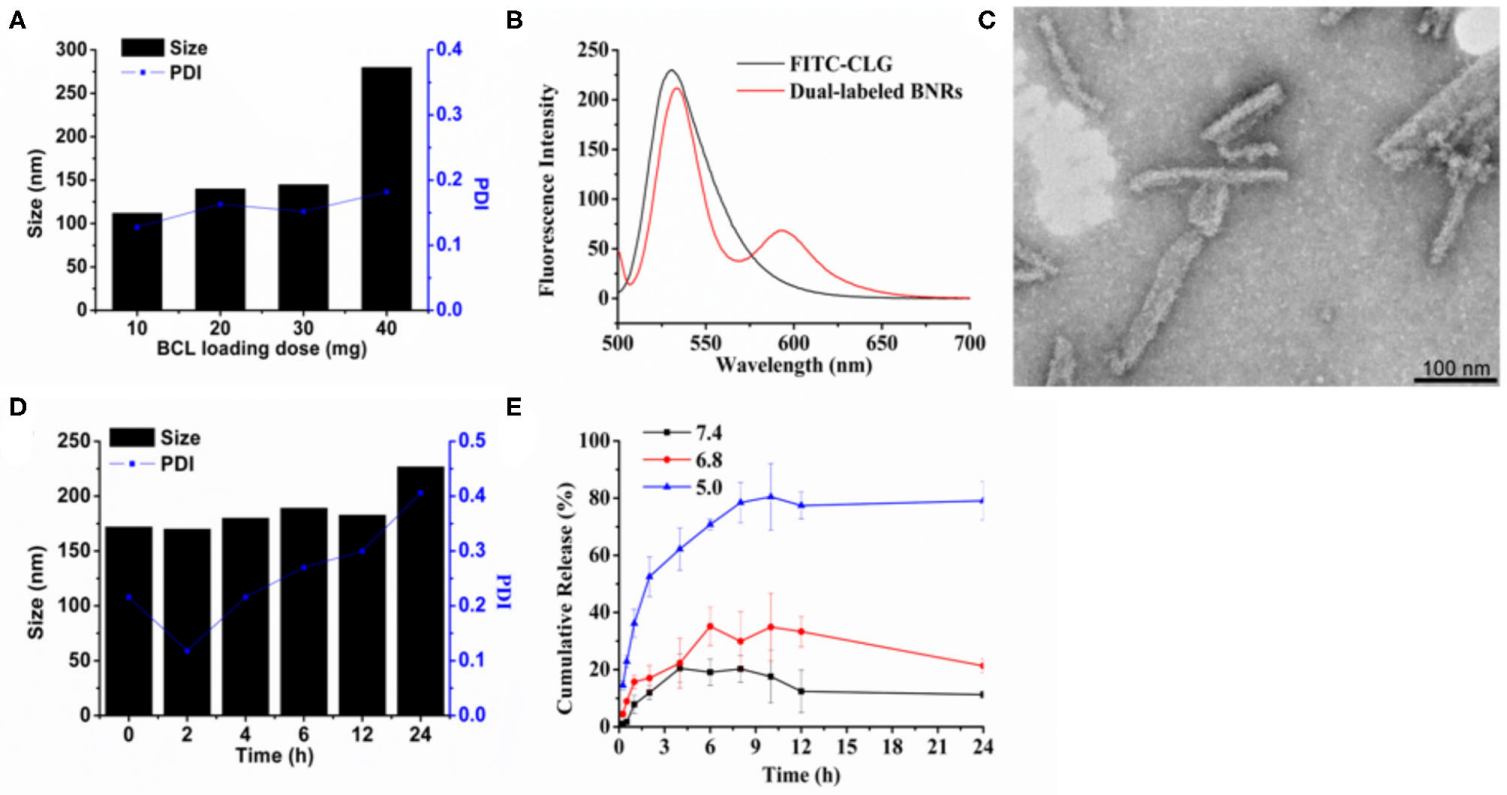
Preparation and characterization. **(A)** Effect of baicalein loading on the particle size of BNRs. **(B)** Fluorescence emission spectra for FRET assay. FITC and RITC were used to label CLG and the drug crystals, respectively. Dual-labeled BNRs were prepared by coating the RITC-labeled drug crystals with FITC-CLG. Excitation wavelength: 492 nm. **(C)** TEM image of optimized formulation (scale bar = 100 nm). **(D)** Particle-size change of BNRs in 10% serum at 37°C for 12 h. **(E)** *in vitro* release of baicalein from BNRs in buffer solution at pH of 5.0, 6.8, and 7.4 at 37°C for 24 h.

CLG is a safe biopolymer and potent to stabilize drug crystals (He et al., 2013; Teng et al., 2020). Herein, BNRs were prepared *via* a precipitation–ultrasonication method by using CLG as a stabilizer. First, the influence of drug loading on the diameter of BNRs was investigated. As displayed in Figure 1A, increasing the drug loading from 10 to 40 mg in a 10 mL CLG solution containing 10 mg CLG resulted in an increase of particle size from 125 to 280 nm. Nonetheless, the diameter was <150 nm as the added drug was <30 mg, and all PDI values are smaller than 0.3, indicating a homogeneous dispersion with narrow size distribution of the nanoparticles. The results demonstrated that the stabilizer CLG is potent to stabilize the drug nanocrystals. To identify the interaction between the drug crystals and CLG, FRET was utilized, in which FITC and RITC were used as the energy donors and acceptors, respectively. FRET effect is displayed *via* the reduction fluorescence intensity of donor at 520 nm with increased fluorescence of the acceptor at 590 nm (Figure 1B), an indicator of the interplay between the stabilizer and the crystals. TEM examination demonstrated that BNRs has a rod shape and diameter of 100–150 nm in length (Figure 1C). The test of serum stability performed *via* incubation in 10% FBS displayed little alternation in the diameter of the nanoparticles at 12 h post-incubation and, as a result, demonstrated the potential stability in physiological conditions (Figure 1D).

The drug release profiles were studied at three pH conditions, pH 5, 6.8, and 7.4, through a dialysis method. The drug release at pHs of 6.8 and 7.4 was <40 and 20% in a 24 h period, respectively, whereas the release at pH 5 was up to 80% (Figure 1E). The results indicated that the drug release from BNRs was pH-dependent due to that BCL is a weak basic drug that has higher solubility in low pH conditions.

### Cellular Uptake *via* Caveolar Pathway

**Figure 2:**
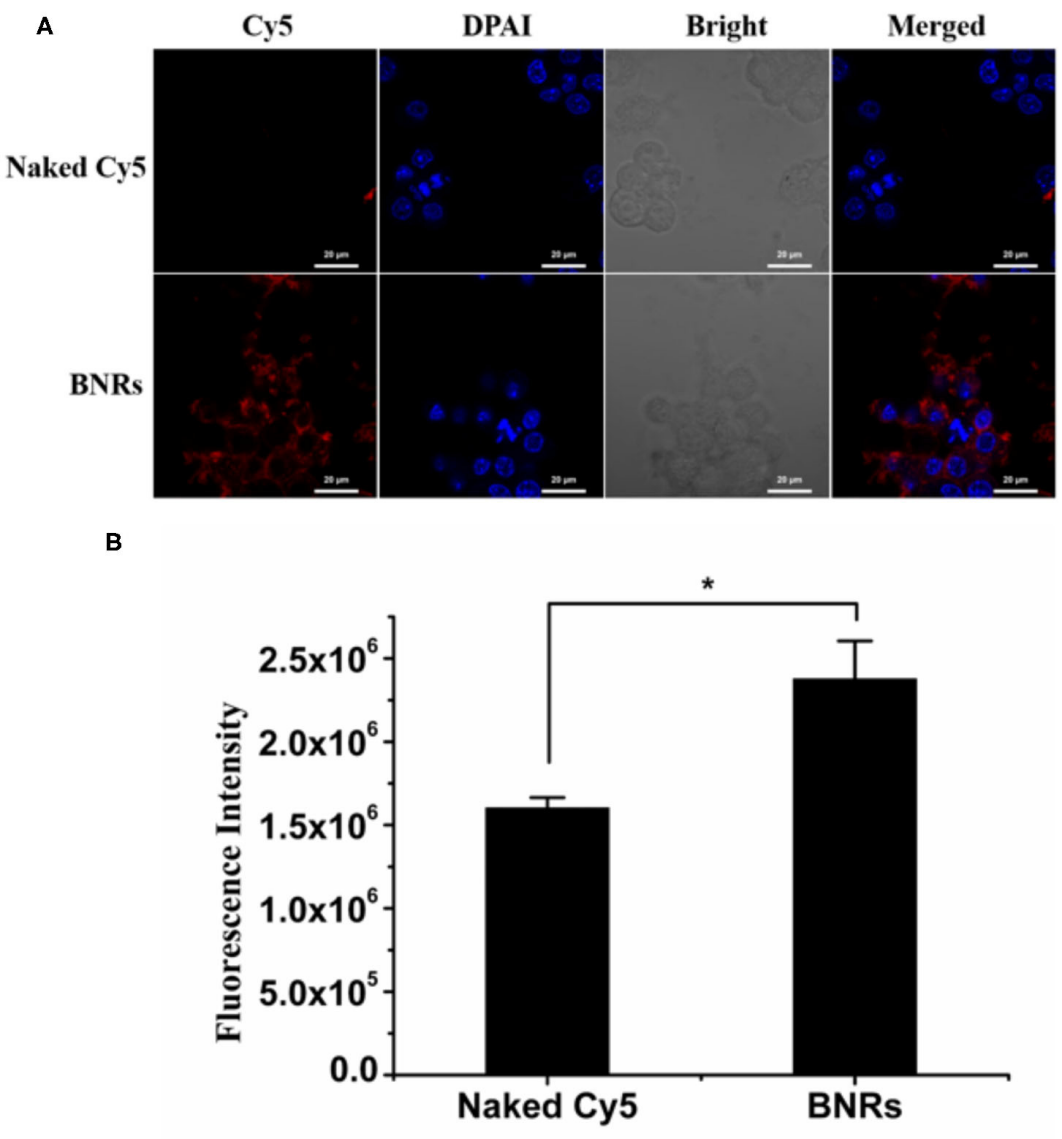
Uptake in macrophages. Uptake of Cy5-labeled BNRs in RAW 264.7 cells was tested by **(A)** CLSM and **(B)** flow cytometry, following incubation at a dye concentration of 100 nM at 37°C for 4 h (mean ± SD, *n* = 3, *P < 0.05). The scale bar is 10 μm.

**Figure 3:**
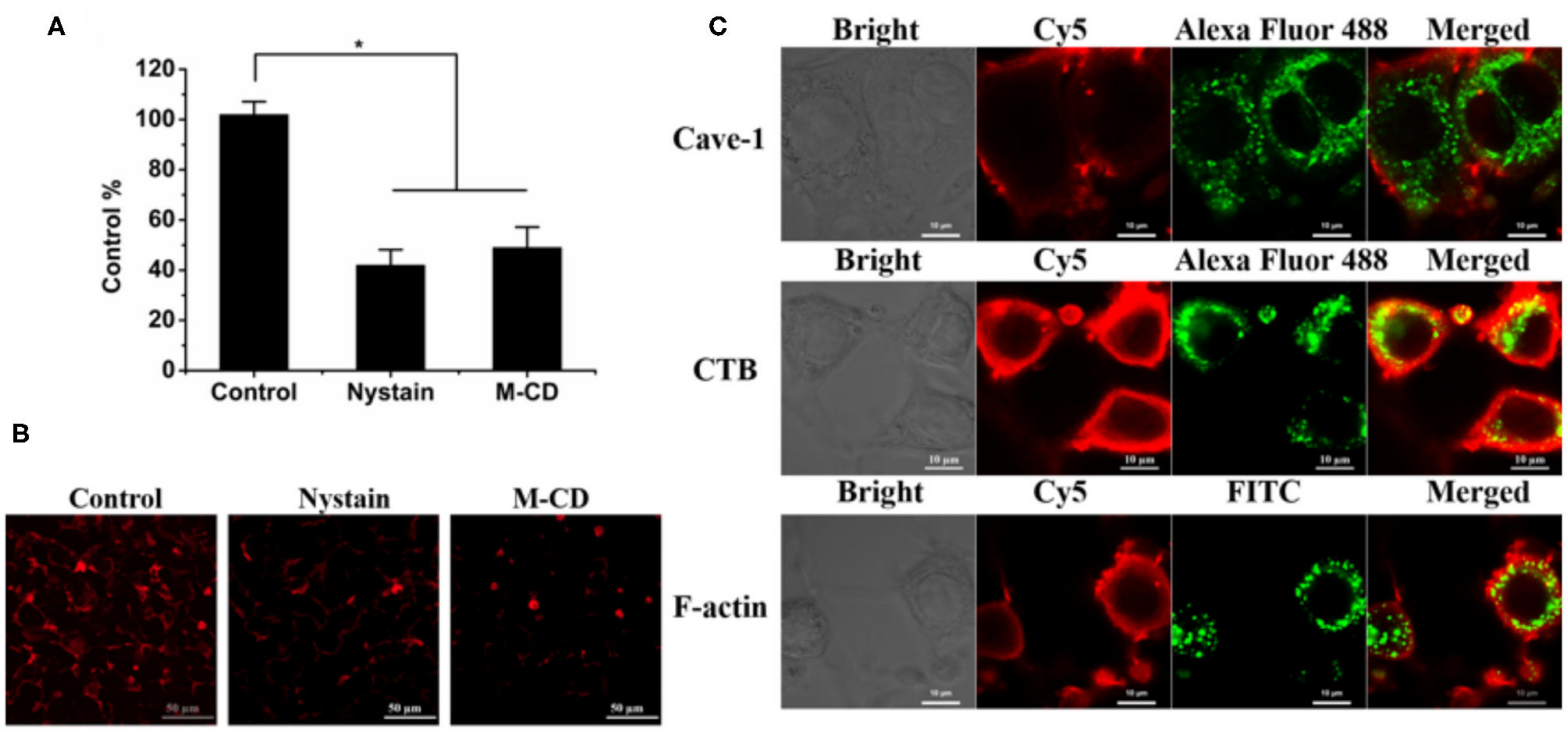
Internalization pathway. **(A,B)** Cellular uptake of Cy5-labeled BNRs in RAW 264.7 cells pre-treated with different inhibitors for 0.5 h was determined by **(A)** flow cytometry (mean ± SD, *n* = 3, *P < 0.01) and **(B)** CLSM observation after a 4 h incubation at 37°C (Scale bar, 50 μm). **(C)** Colocalization of Cy5-labeled BNRs with caveolae-trafficking proteins, Alexa Fluor 488-labeled CTB (green), Alexa Fluor 488-labeled Cave-1 (green), and FITC-labeled F-actin (green). The scale bar is 10 μm.

First, the uptake in macrophages was evaluated. After a 4 h incubation, strong red fluorescent spots around the nucleic were displayed (Figure 2A), confirmed by the determination by flow cytometry (Figure 2B). Second, previous reports demonstrated that rod-like particles with a diameter of <200 nm in length always obtained cellular entry *via* the caveolar pathway, bypassing the endo-lysosomal systems (Xin et al., 2017, 2018, 2019). As depicted in Figure 3A, the internalization of BNRs decreased by ~60% in the cells pre-treated with inhibitors of caveolar pathway, nystatin, or M-CD, compared with the control pre-treated with saline. The reduced uptake was verified by the examination by fluorescence microscopy (Figure 3B). In order to confirm the participation of the caveolar pathway in the uptake, co-localization of Cy5-labeled BNRs with caveolae-trafficking proteins, Cav-1, CTB, and F-actin, was studied using CLSM. Yellow fluorescent spots that appeared in the merged pictures demonstrated the colocalization of the nanoparticles with the three proteins (Figure 3C). These results indicated that 150 nm BNRs entered cells mainly *via* the caveolar pathway.

### Enhanced Phenotype Switch From M1 to M2 and Anti-inflammation

**Figure 4:**
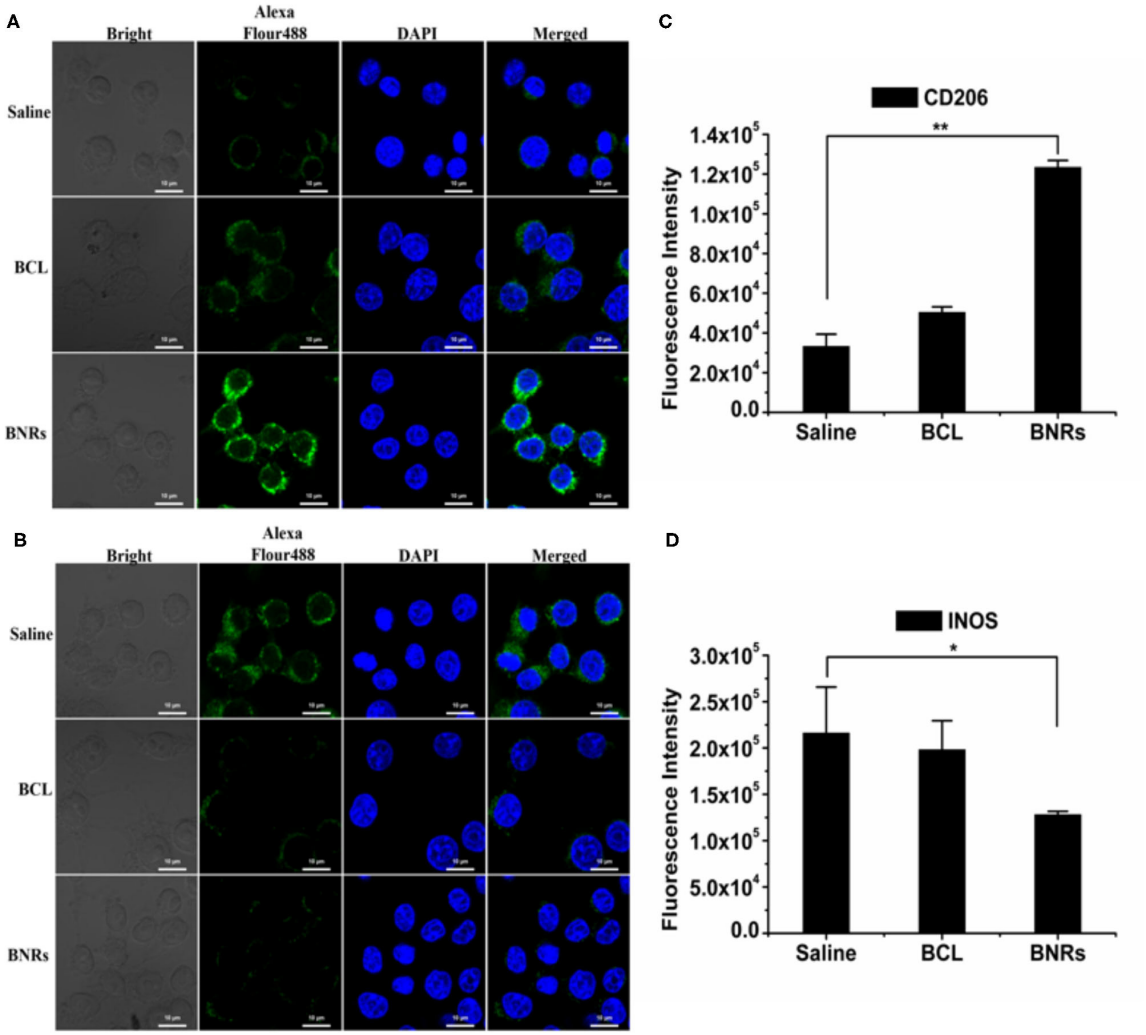
Phenotypic shift study *in vitro*. Immunofluorescence and quantitative analysis of CD206 **(A,C)** and INOS **(B,D)** expression on RAW 264.7 cells after treatment (*n* = 3, *P < 0.05, and **P < 0.01). The nuclei were stained with DAPI (blue) and CD206 and INOS were stained with Alexa Fluor 488-conjugated antibody (green). The scale bar is 10 μm.

**Figure 5:**
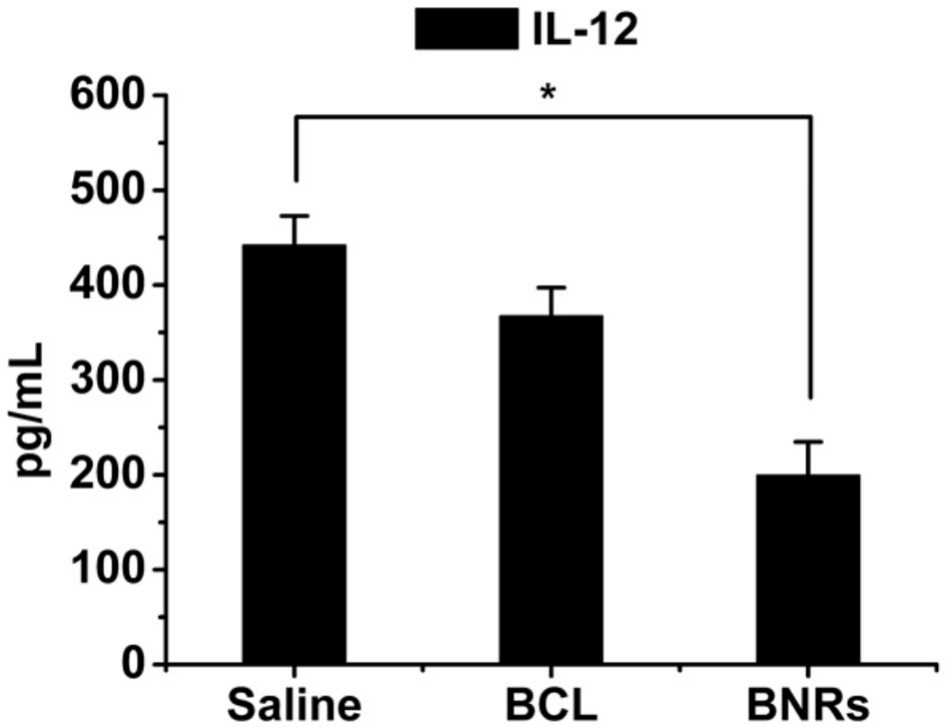
Anti-inflammation *in vitro*. Quantitative analysis of IL-12 expression on RAW 264.7 cells after treatment for 12 h (*n* = 3, *P < 0.05).

BCL is potent to alleviate anti-inflammation effects. In this study, we hypothesized that BNRs allowed for reduced inflammation activities *via* promoting the phenotypic switch of macrophages from proinflammatory phenotype (M1) to anti-inflammatory phenotype (M2). To characterize the polarization of macrophages, two markers, CD206 and INOS, that highly express on phenotypic M2 and M1, respectively (He W. et al., 2019), were detected by immunofluorescence assay. Treatment with free BCL or BNRs upregulated CD206 compared with saline (Figure 4A), confirmed by the quantified results assayed by flow cytometry (Figure 4B). Importantly, BNRs enabled an ~2-fold increase in the expression of CD206 over free BCL. Also, treatment with BNRs reduced the expression of INOS significantly (Figures 4C,D). Next, the anti-inflammatory effect was assessed in macrophages after incubation with various formulations. As expected, dosing BNRs significantly reduced the secretion of inflammatory factor, IL-12, along with a >50% decrease with that from BCL (Figure 5). The results demonstrated that BNRs polarized M1 into M2 with high efficacy and leased the inflammation effectively.

The enhanced anti-inflammation effect *in vitro* resulted from the benefits of BNRs, high drug-loading capacity, and sustained drug release. To react to the surrounding situations, macrophages tend to alter their polarization, implying that the polarization may be changed once the drug's simulation disappears (He W. et al., 2019). As is well-known, most of drug delivery systems (DDS) release their drugs rapidly in cells after uptake due to the decomposition by the endo-lysosomes (He et al., 2020), always discounting the inflammatory activity of drugs. Previous reports indicated that the drug crystals in cells could keep their integrity up to 7–10 h (Lu et al., 2017; Qi et al., 2019). In this study, the BNRs with a payload capacity of 7–10-fold increase over conventional DDS entered cells *via* bypassing the endo-lysosomes and, as a result, maintained their integrity in the cytosol. These profiles allowed BNRs to sustain release of BCL over time after uptake. Indeed, as displayed in Figure 1E, BNRs released the drug in a slow pattern. Overall, the nanocrystal approach is promising to improve the efficacy of anti-inflammatory drugs against inflammatory diseases such as myocarditis, atherosclerosis, pulmonary hypertension, stroke, and cardiac disease.

### Biocompatibility Study

**Figure 6:**
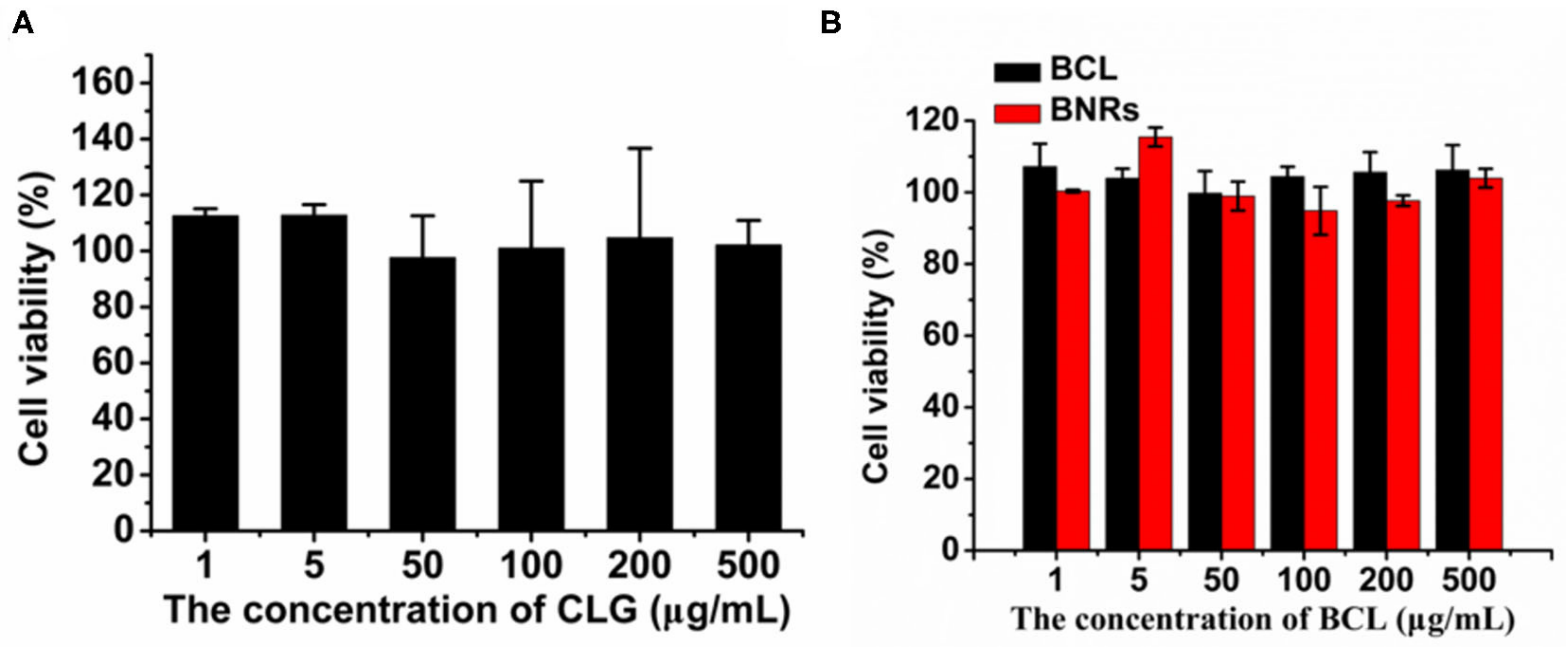
Cell viability. Cytotoxicity of **(A)** stabilizer CLG and **(B)** formulations to RAW 264.7 cells post 48 h incubation.

**Figure 7:**
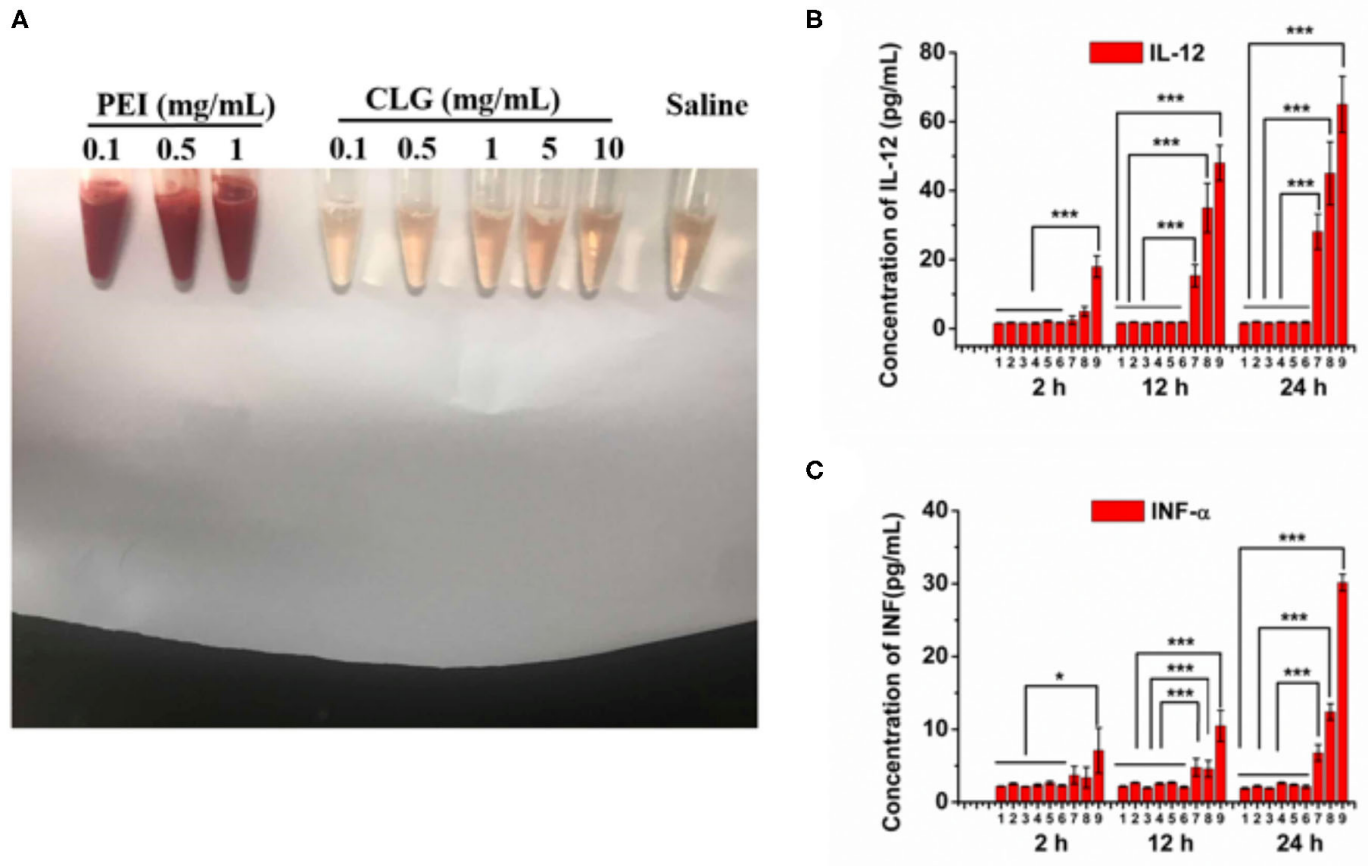
Biocompatibility. **(A)** Hemolytic activity of CLG (0.1–10 mg/mL). A cationic vector, PEI, was used as a positive control. Inflammatory factors, **(B)** IL-12, and **(C)** INF-α, in the mouse plasma after injection of CLG or PEI for 2, 12, and 24 h, respectively. The formulations and administration doses based on the body weight are as follows: 1, saline; 2, CLG, 1 mg/kg; 3, CLG, 5 mg/kg; 4, CLG, 10 mg/kg; 5, CLG, 50 mg/kg; 6, CLG, 100 mg/kg; 7, PEI, 1 mg/kg; 8, PEI, 2.5 mg/kg; 9, PEI, 5 mg/kg. *n* = 3, *P < 0.05 and ***P < 0.001.

First, the cell viability of the stabilizer CLG used in the formulations and BNRs was determined by the MTT assay. CLG and BNRs have little cytotoxicity to macrophages in the tested concentrations (Figure 6). Then, hemolytic activity was investigated by using PEI, a cationic vector, as positive control. Incubation with PEI resulted in profound hemolysis. In contrast, treatment with CLG at pre-determined concentrations showed ignored hemolytic toxicity (Figure 7A). In addition, the immunogenicity was evaluated by determination of inflammatory factors, IL-12 and INF-α, in the plasma after intravenous injection of CLG or PEI at specific doses. The levels of the two factors from the groups dosed with PEI went up markedly at 12 or 24 h post-injection compared with that from the saline-treated group (Figures 7B,C), whereas the injection of CLG did not increase the concentration of the factors. The results indicated that CLG is safe for intravenous injection.

## Conclusions

In this study, BCL nanocrystals were prepared and characterized. The nanocrystals have a diameter of ~150 nm with a rod-like structure. *Via* internalization through the caveolar pathway, the drug nanocrystals are potent to promote the phenotypic switch from proinflammatory M1 to anti-inflammatory M2 and, as a result, alleviate the inflammation activity *in vitro*. In conclusion, crystallization is a promising strategy to improve the activity of insoluble anti-inflammatory agents.

## Data Availability Statement

All datasets presented in this study are included in the article.

## Ethics Statement

The animal study was reviewed and approved by The China Pharmaceutical University Institutional Animal Care and Use Committee. Written informed consent was obtained from the owners for the participation of their animals in this study.

## Author Contributions

WH conceived and designed the research work. JZ, CT, and CL performed the experiments. All of the authors discussed the results and commented on the manuscript. All of the authors have read and approved the final manuscript.

## Conflict of Interest

The authors declare that the research was conducted in the absence of any commercial or financial relationships that could be construed as a potential conflict of interest.
